# Tick feeding modulates the human skin immune landscape to facilitate tick-borne pathogen transmission

**DOI:** 10.1172/JCI161188

**Published:** 2022-11-01

**Authors:** Johanna Strobl, Verena Mündler, Sophie Müller, Anna Gindl, Sara Berent, Anna-Margarita Schötta, Lisa Kleissl, Clement Staud, Anna Redl, Luisa Unterluggauer, Ana E. Aguilar González, Sophie T. Weninger, Denise Atzmüller, Romana Klasinc, Gerold Stanek, Mateusz Markowicz, Hannes Stockinger, Georg Stary

**Affiliations:** 1 Department of Dermatology, Medical University of Vienna, Vienna, Austria.; 2 CeMM Research Center for Molecular Medicine of the Austrian Academy of Sciences, Vienna, Austria.; 3 Institute for Hygiene and Applied Immunology, Center for Pathophysiology, Infectiology and Immunology, Medical University of Vienna, Vienna, Austria.; 4 Ludwig Boltzmann Institute for Rare and Undiagnosed Diseases, Vienna, Austria.; 5 Department of Plastic and Reconstructive Surgery, Medical University of Vienna, Vienna, Austria.; 6 Austrian Agency for Health and Food Safety (AGES), Vienna, Austria.

**Keywords:** Immunology, Adaptive immunity, Skin

## Abstract

During cutaneous tick attachment, the feeding cavity becomes a site of transmission for tick salivary compounds and tick-borne pathogens. However, the immunological consequences of tick feeding for human skin remain unclear. Here, we assessed human skin and blood samples upon tick bite and developed a human skin explant model mimicking *Ixodes ricinus* bites and tick-borne pathogen infection. Following tick attachment, we observed rapidly occurring patterns of immunomodulation, including increases in neutrophils and cutaneous B and T cells. T cells upregulated tissue residency markers, while lymphocytic cytokine production was impaired. In early stages of *Borrelia burgdorferi* model infections, we detected strain-specific immune responses and close spatial relationships between macrophages and spirochetes. Preincubation of spirochetes with tick salivary gland extracts hampered accumulation of immune cells and increased spirochete loads. Collectively, we showed that tick feeding exerts profound changes on the skin immune network that interfere with the primary response against tick-borne pathogens.

## Introduction

Ticks are hematophagous ectoparasites, with the skin representing the main interface of host-vector interaction that may become the site of tick-borne pathogen transmission. Steadily increasing tick prevalence has been reported globally in recent years, a trend that is predicted to increase with the rise in global temperatures and consequently more temperate climate (1). Milder winters contribute to changes in wildlife-host populations, as well as shortening of the previously naturally occurring breaks in tick activity, resulting in a consistent increase in tick bite–associated (TB-associated) health complications annually (2). Human hosts are affected primarily by diseases spread by the various species of the hard-tick family (*Ixodidae*, genus *Ixodes*) distributed worldwide. Since the summer of 2019, the European Centre for Disease Prevention and Control reported 7,563 novel locations in addition to previously known territorial distribution of *I*. *ricinus* species, and modeling data suggest a continuous further spread of this tick species to higher altitudes and latitudes (3).

Each year, thousands of patients affected by tick-borne diseases are admitted to hospitals around the world and there is still a significant gap in effective diagnosis, targeted treatment, or sufficient preventative measures. *I*. *ricinus* has been identified as a transmission vector of a high diversity of pathogens, such as tick-borne encephalitis virus (4, 5) and *Borrelia* *burgdorferi* sensu lato (*Bbsl*) that causes Lyme borreliosis in Europe, with an estimate of 85,000 to 230,000 cases annually (6, 7).

*Bbsl*, among other pathogens transmitted by *Ixodes* ticks, has been shown to use tick salivary compounds to perfect its mode of transmission and ability to infect human hosts (8, 9). Tick saliva contains a wide variety of effector molecules (10, 11) acting in a cytolytic, anticoagulant (syntaxin, synaptobrevin, SNAP-35 complex) (12), antiinflammatory (Salp15, ISL929, ISL1373) (13), antichemokine (evasins) (14), antipain and vasodilating (prostaglandin E2) (15) manner, and aiding tick congregation during transmission (ixofin3D) (16), making ticks truly remarkable disease vectors. Many of these act with high specificity toward the immune system: Salp15 has been shown to specifically repress CD4^+^ T cell activation upon binding to CD4 coreceptors (13, 17); prostaglandin E2 can inhibit dendritic cell (DC) maturation and function (18); and evasin-1 and -3 affect multiple cell types, targeting key inflammatory cytokines and chemokines, such as CCL3, CXCL8, CXCL1, or CCL5 (19). On a cellular level, Krause et al. reported decreased inflammatory infiltrate at the site of *Ixodes* *scapularis* attachment to skin of BALB/c mice and 4 human donors (20). The evolutionary function of tick saliva may be to inhibit various defense lines that the host mounts in response to skin injury and TB. However, microbes such as *Bbsl* successfully coevolved to use them to their advantage during saliva-assisted transmission (21). *Bbsl* spirochetes can persist in the tick’s gastrointestinal system by binding the tick midgut protein TROSPA and move through the tick’s hemolymph to the salivary glands upon initiation of blood feeding (22). There, *Bbsl* can bind to Salp15 via its outer surface lipoprotein C (OspC), which allows it to travel into the host and replicate, protected from complement-mediated killing and antibody response (23).

Indeed, the immunosuppressive effects of several tick salivary compounds are well established. However, many of the consequences of tick feeding and its associated barrier disruption for the local and circulating host immune system remain elusive due to lack of model systems and studies in human skin (24). To our knowledge, this is the first systematic investigation of immune cells at human tick feeding sites and in blood samples. Furthermore, the present study investigates initial events at the host-pathogen interface in a human ex vivo model system mimicking TB.

## Results

### Analysis of tick feeding sites reveals a pattern of immunomodulation in human skin.

**Figure 1:**
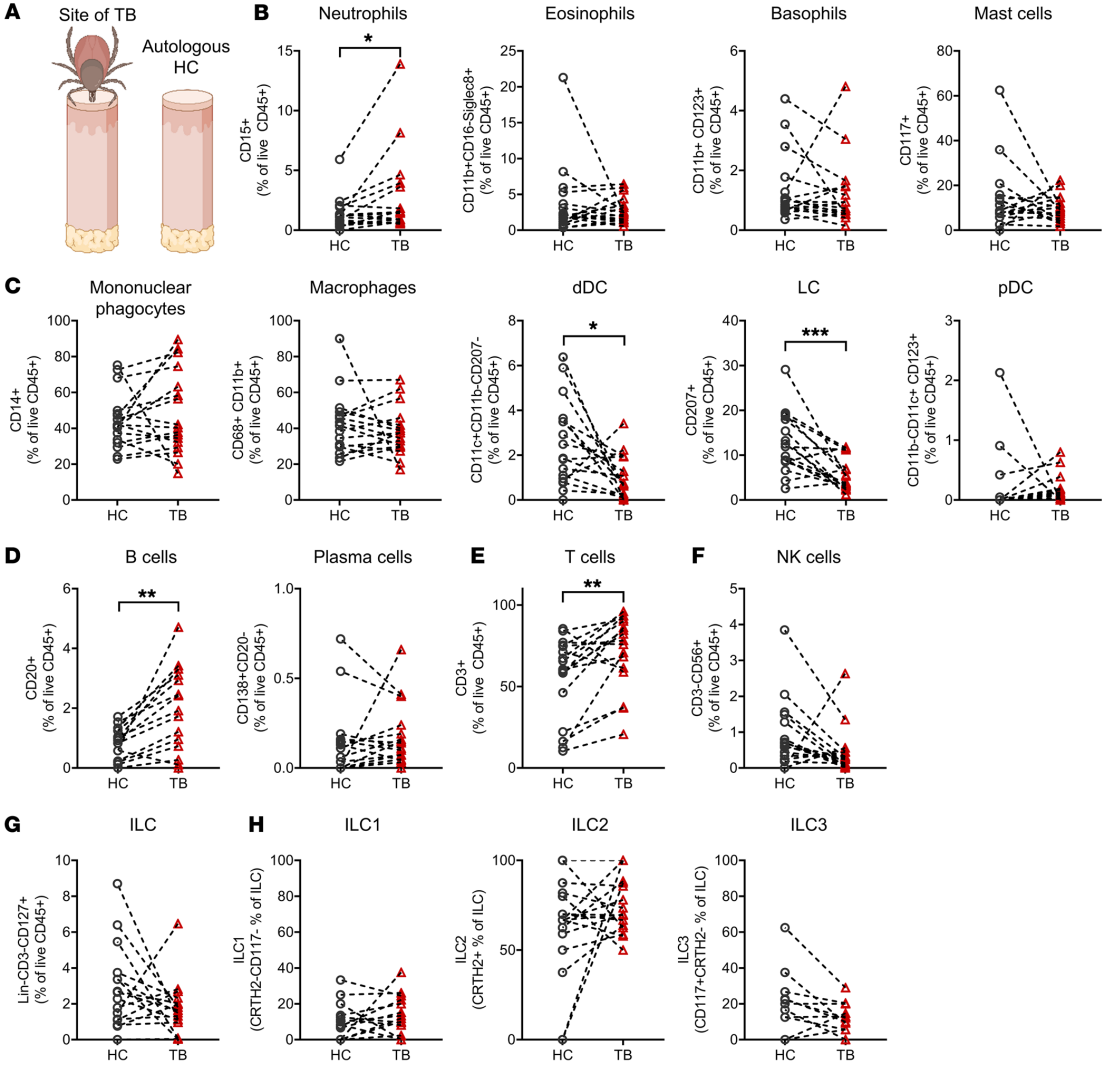
Skin biopsies of tick feeding sites show changes in the immune cell composition in human skin. (**A**) Illustration of sampling sites. (**B**–**H**) Percentages of neutrophils, eosinophils, basophils, mast cells (**B**), mononuclear phagocytes, macrophages, dDCs, LCs, pDCs (**C**), B cells, plasma cells (**D**), T cells (**E**), NK cells (**F**), and ILCs (**G**) among live cells and ILC subsets (**H**) in TB and autologous HC. Data shown as percentage of live CD45^+^ cells (**B**–**G**) and percentage of CRTH2^–^CD117^–^ (ILC1), CRTH2^+^CD117^–^ (ILC2), and CRTH2^–^CD117^+^ (ILC3) (**H**) among ILCs in TB and HC. In **A**–**H**, 1 dot represents one patient, and dotted lines connect interindividual samples (*n =* 16). Statistical analysis was performed by paired Student’s *t* test. *P < 0.05, **P < 0.01, ***P < 0.001.

To analyze the direct effects of tick feeding on human immune cells, we recruited healthy individuals after TBs and assessed leukocyte populations of the peripheral blood, skin at the TB site, and healthy skin from the same individuals in parallel. One out of 19 skin biopsies tested PCR positive for *Bbsl* (strain *B*. *afzelii*) and was therefore excluded from subsequent analysis (Table 1). Comparing leukocyte suspensions isolated from TB skin to those isolated from nonaffected healthy-appearing skin (healthy control, HC) of the same individual by flow cytometry (Figure 1A, gating strategy depicted in Supplemental Figure 1A; supplemental material available online with this article; https://doi.org/10.1172/JCI161188DS1), we detected a distinct pattern of immunomodulation at the tick feeding site. We found significantly higher frequencies of neutrophils in TB skin, indicating a proinflammatory response (Figure 1B). Eosinophil, basophil, and mast cell frequencies were comparable to HC skin.

A decreased number of Langerhans cells (LCs) has been described in cutaneous Lyme borreliosis, the most common tick-borne skin infection (25, 26). However, it remains unknown whether this effect is mediated by *Borrelia* infection or tick feeding and associated tick salivary products. We therefore investigated the frequencies of cutaneous antigen-presenting cell subsets and detected decreased frequencies of CD207^+^ LCs and CD11c^+^CD11b^–^CD14^–^ dermal DCs (dDCs). Frequencies of CD14^+^ mononuclear phagocytes, CD11b^+^CD68^+^ macrophages, and CD11b^–^CD123^+^ plasmacytoid DCs (pDCs) remained unchanged (Figure 1C). Further diagnostic features of early *Borrelia* skin infection are increased cutaneous B cell numbers and a plasma cell infiltrate (27). Interestingly, we detected significantly increased B cell frequencies in the skin after a TB without *Borrelia* transmission, while plasma cell levels remained unchanged (Figure 1D). Next, we analyzed T cell numbers and found increased frequencies of CD3^+^ lymphocytes in TB skin (Figure 1E), but did not detect significant changes in frequencies of other lymphocyte populations, including NK cells and innate lymphoid cell (ILC) subsets (Figure 1, F and G). For deeper understanding of time-dependent changes in the immune cell network, we subdivided samples into 3 categories depending on the duration between anamnestic TB and tissue sampling (≤24 hours, *n =* 4; 2–4 days, *n =* 6; 5–7 days, *n =* 6; Supplemental Figure 1B). We found that trends of observed effects, including T cell infiltration and decreases in LCs and dDCs, were detectable early (≤24 hours) after TB. To dissect immunomodulatory patterns induced by tick feeding and those mediated by mere barrier disruption, we performed ex vivo wounding of human skin explants (Supplemental Figure 2A). Compared with HC skin of the same individual, artificial sterile puncture wounds induced an influx of neutrophils and NK cells to the puncture site (Supplemental Figure 2, B and C). In addition, we observed a decrease in ILCs at puncture sites and a shift from ILC2 predominance in HC to ILC1/3 in wounded skin (Supplemental Figure 2C).

Overall, our observations indicate rapid implications of tick feeding for the cutaneous immune cell network, which are distinct from those mediated by skin punctures and include local neutrophil and lymphocytic inflammation and a decrease in local antigen-presenting cells despite the absence of tick-borne pathogens.

### TB immunomodulation elicits a systemic effect on blood lymphocytes.

**Figure 2:**
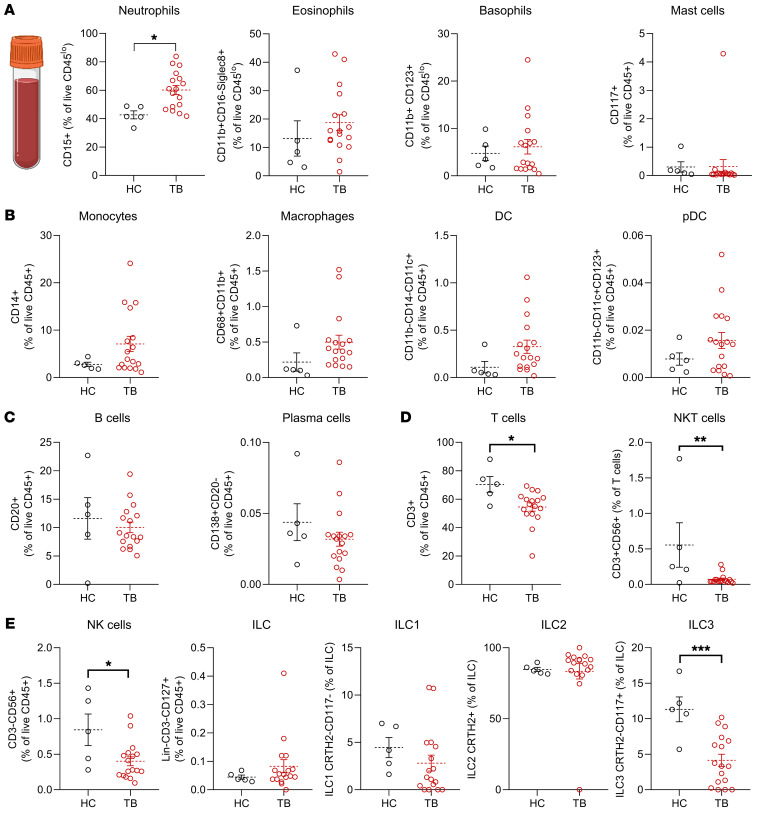
Peripheral blood lymphocyte populations in individuals after tick bite differ from those of healthy individuals. (**A**–**E**) Percentages of neutrophils, monocytes, eosinophils, basophils, mast cells (**A**), DCs, monocytes, macrophages, pDCs (**B**), B cells, plasma cells (**C**), T cells (**D**), and CD56^+^CD3^–^ NK cells and CD127^+^Lineage^–^CD3^–^ ILCs (**E**) among live CD45^+^ cells in blood of individuals affected by TB and HC. Data shown as mean percentage of live CD45^+^ cells, except percentages of CRTH2^–^CD117^–^ (ILC1), CRTH2^+^CD117^–^ (ILC2), and CRTH2^–^CD117^+^ (ILC3) (**E**) among ILCs in blood from TB and HC. In **A**–**E**, 1 dot represents 1 patient (TB, *n =* 16; HC, *n =* 5). Error bars indicate SEM. Statistical analysis was performed by unpaired Student’s *t* test. *P < 0.05, **P < 0.01, ***P < 0.001.

To investigate potential effects on circulating leukocyte populations, we sampled peripheral blood of the same individuals after TB and compared cell surface marker distribution to that of healthy, TB-naive persons. As expected of a locally restricted inflammatory process, we did not observe any changes in peripheral blood granulocytes (Figure 2A) or mononuclear phagocytes (Figure 2B, gating strategy shown in Supplemental Figure 3A). Blood levels of B cells and plasma cells were not significantly altered in individuals after TB (Figure 2C). Interestingly, we detected a significant decrease in circulating CD3^+^ T cells, NK cells, and NK T cells upon TB (Figure 2, D and E). Furthermore, CD117^+^ type 3 ILCs (ILC3s), a key sentinel cell type in tissue homeostasis that rapidly responds to barrier disruption (28), were present in lower frequencies in peripheral blood upon TB compared with healthy individuals (Figure 2E). Cellular differences in TB-affected individuals were conserved when we corrected for age (Supplemental Figure 3B). Overall, our observations indicate that tick feeding elicits pronounced changes in the local skin immune system, but may also confer subtle systemic effects on adaptive and innate lymphocytes in the peripheral blood.

### Impaired T cell and ILC responses after TB.

**Figure 3:**
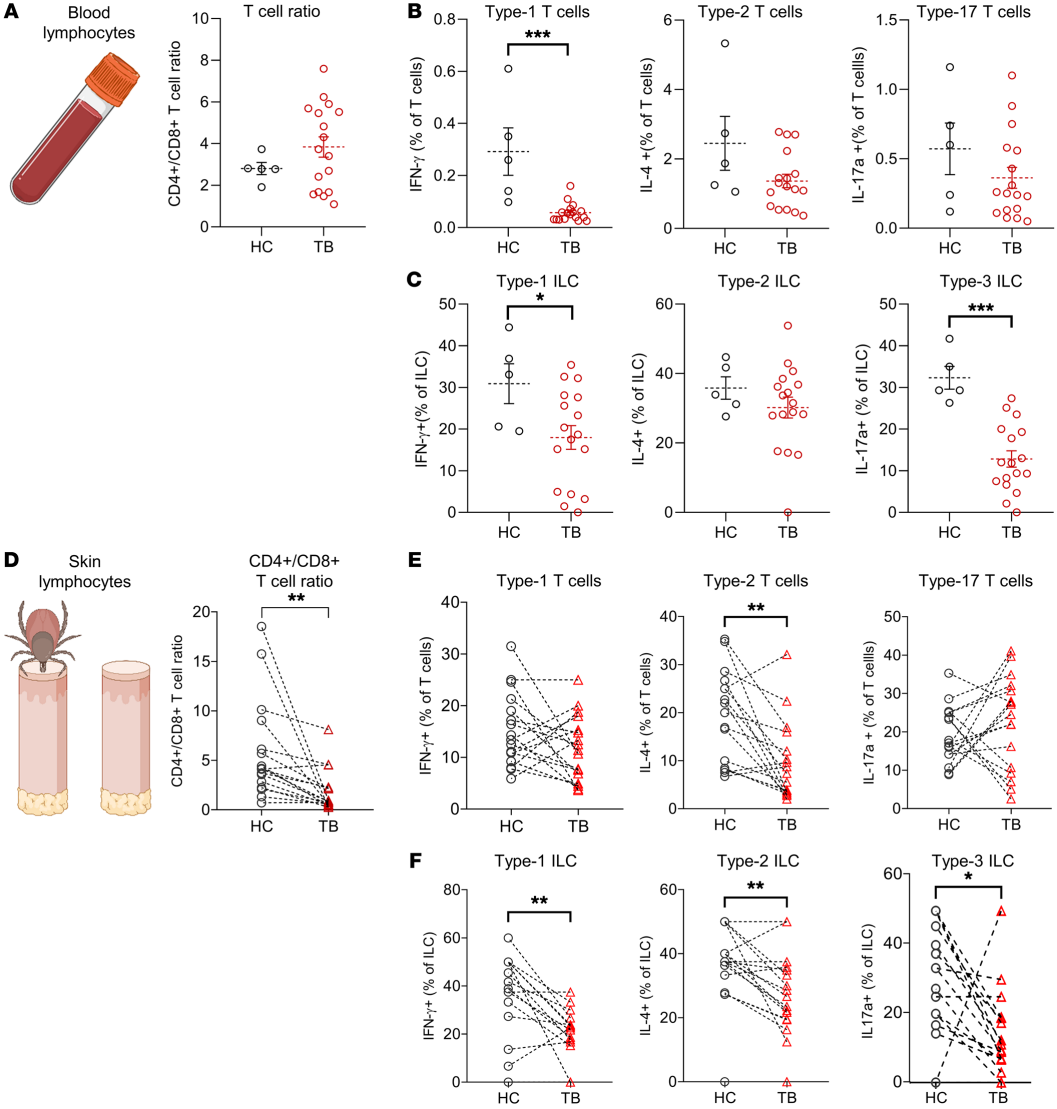
Intracellular cytokine staining reveals impaired T cell and ILC responses in skin and blood after tick bite. (**A**) Ratio of CD4^+^/CD8^+^ T cells among T cells isolated from blood of TB (*n =* 16) and HC (*n =* 5). (**B** and **C**) Frequencies of cells expressing IL-4, IL-17a, and IFN-γ among T cells (**B**) and ILCs (**C**) in blood from TB (*n =* 16) and HC (*n =* 5) upon stimulation with PMA and ionomycin. (**D**) Ratio of CD4^+^/CD8^+^ T cells among skin T cells isolated from the site of TB and autologous HC skin (*n =* 16). (**E** and **F**) Frequencies of cells expressing IL-4, IL-17a, and IFN-γ among T cells (**E**) and ILCs (**F**) in TB skin and autologous HC (*n =* 16) upon stimulation with PMA and ionomycin. One symbol represents one patient, and dotted lines connect intraindividual samples. Data are presented as mean ± SEM. Statistical analysis was performed with unpaired (**A**–**C**) or paired (**D**–**F**) Student’s *t* test. *P < 0.05, **P < 0.01, ***P < 0.001.

Several compounds of tick saliva have been implicated in potential suppression of T cell function. Therefore, we isolated T cells and ILCs from skin and peripheral blood of individuals after TB, stimulated them with a cell activation cocktail containing PMA, ionomycin, and brefeldin A, and performed intracellular cytokine labeling after cell permeabilization (gating strategy depicted in Supplemental Figure 4, A and B). Although we had detected increased T cell frequencies in TB skin samples, we detected unchanged ratios of peripheral blood CD4^+^ to CD8^+^ T cells (Figure 3A), signifying a simultaneous increase in both T cell subsets. When we analyzed the functionality of T helper cell subsets, we found decreased capacities to produce the type 1 cytokine IFN-γ (Figure 3B). Frequencies of type 2 cytokine IL-4–producing and type 17 cytokine IL-17A–producing peripheral blood T cells were also decreased in some individuals. However, this effect was not significant. We next investigated production of cytokines by circulating ILCs and saw similar effects, with significantly decreased capacities for type 1 and type 17 cytokine production (Figure 3C).

In TB skin lesions, we detected a significant decrease in CD4^+^ to CD8^+^ T cell ratios, signifying an overall rise in CD8^+^ T cell numbers (Figure 1E and Figure 3D). Cytokine production by cutaneous lymphocytes was similar to blood-derived lymphocytes, with a trend of decreased IFN-γ–producing (type 1) T cells and IL-17A–producing ILCs (Figure 3, E and F). Interestingly, cutaneous type 2 responses were also significantly reduced in skin upon TB. This is in contrast to findings in mice, where tick feeding induced the capacity for IL-4 production (29) and Th2 responses were associated with decreased bacterial load upon infection with *B*. *burgdorferi* sensu stricto (*Bbss*) (30). Overall, we detected impaired functions of both skin and peripheral blood T helper and ILC subsets in regard to their cytokine production capacities, which has important implications for cutaneous immunity to tick-borne pathogens.

### Tissue-resident memory T cells and γδ T cells are induced by TB.

Tissue-resident memory T cells (TRMs) are at the forefront of barrier defense, providing rapid recall responses to invading pathogens. Besides antibody-mediated immunity, a robust T cell response is crucial for long-lasting immunity induced by vaccine candidates against tick-borne infections. We therefore investigated T cells and their expression of human TRM markers (31) CD69 and integrin αE (CD103) in TB-affected skin sections by fluorescence microscopy.

**Figure 4:**
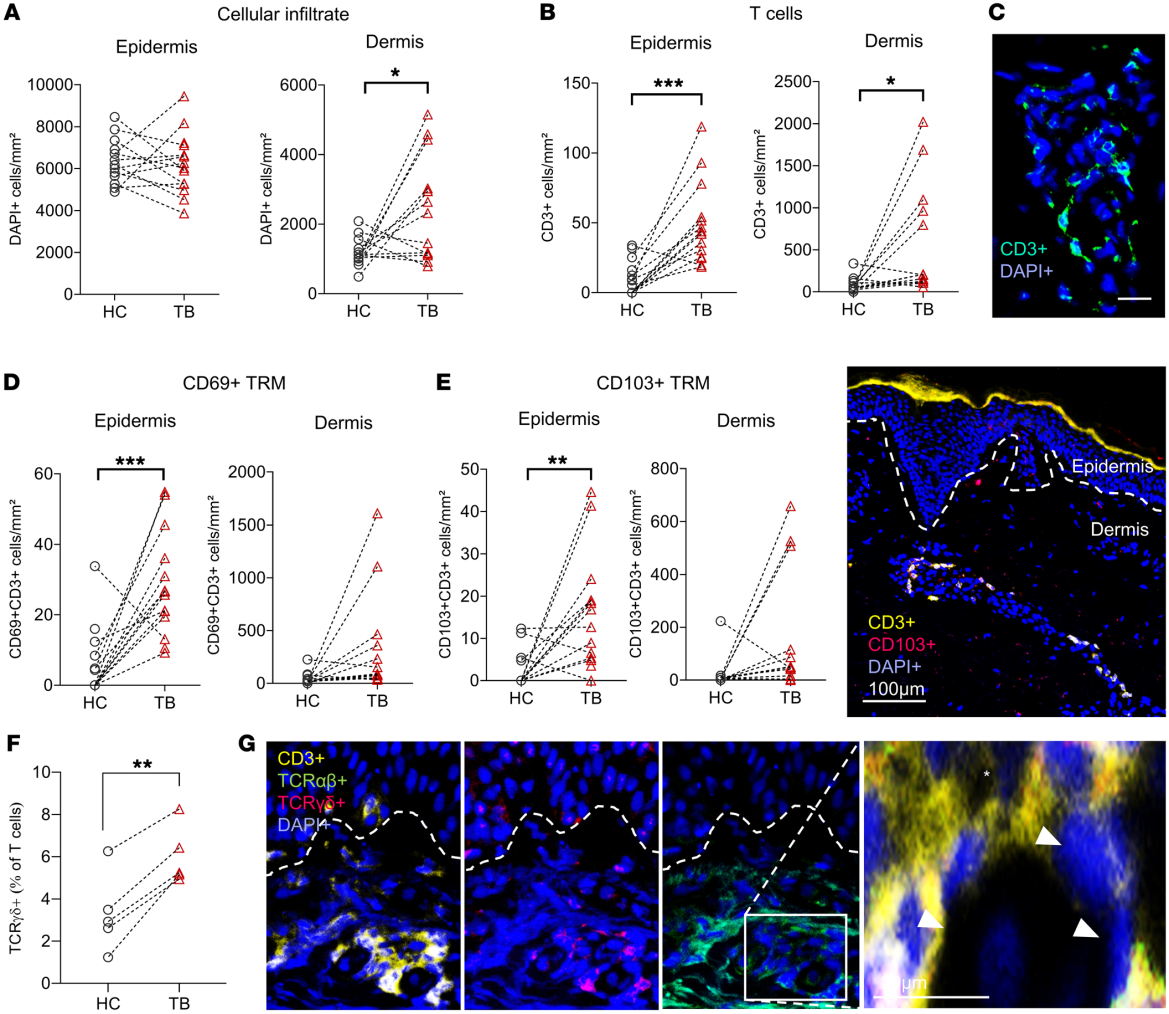
Skin sections of clinical and experimental tick bites harbor increased numbers of tissue-resident memory T cells. (**A**–**C**) Lymphocytic infiltrate in TB (*n =* 11) and intraindividual HC samples (*n =* 11) as determined by immunolabeling of DAPI^+^ cells (**A**) and CD3 (**B**). Data shown as cell number per mm^2^ in dermis and epidermis. (**C**) Representative image of a CD3^+^ and DAPI-counterstained TB skin sample. Scale bar: 20 μm. (**D** and **E**) TRMs in TB (*n =* 11) and intraindividual HC samples (*n =* 11), CD69^+^ T cells (**D**), and CD103^+^ T cells (**E**). Data shown as cell number per mm^2^ in dermis and epidermis. (**E**) Right panel: Representative image of dermal and epidermal TRMs in TB skin. Scale bar: 100 μm. (**F**) Quantification of TCRγδ^+^ T cells in HC and TB skin (*n =* 4). Data shown as percentage of T cells (CD3^+^). (**G**) Representative image of DAPI, CD3, TCRαβ, and TCRγδ immunofluorescence staining (×20 magnification, left panels) in TB and HC skin and magnification (right panel). Arrows indicate TCRγδ^+^ T cells and asterisk shows TCRαβ positivity. Scale bar: 20 μm. In **A**–**F**, statistical analysis was performed using paired Student’s *t* test. **P < 0.01, ***P < 0.001.

Increased cellular infiltration — as measured by number of DAPI^+^ cells per mm² skin compared to autologous HC skin — was observed only in the dermal and not epidermal regions of TB-affected skin (Figure 4A), which may be attributed to higher baseline cellularity and stability of structural cells in the epidermis. T cell numbers increased in the epidermis and dermis (Figure 4, B and C), in line with our analysis by flow cytometry (Figure 1E). Furthermore, we saw an increase in CD69^+^, CD103^+^, and CD69^+^CD103^+^ double-positive TRMs in the epidermal compartment (Figure 4, D and E, and Supplemental Figure 5A). While the vast majority of skin TRMs expresses the αβ T cell receptor (TCRαβ), cells expressing the TCRγδ have been documented to play a key role in immune surveillance of barrier tissues (32). We therefore investigated the distribution of TCRαβ and TCRγδ in TB and HC samples and found increased percentages of TCRγδ^+^ T cells in TB skin (Figure 4, F and G).

### Injection of tick salivary gland extracts mimics tick feeding on human skin.

**Figure 5:**
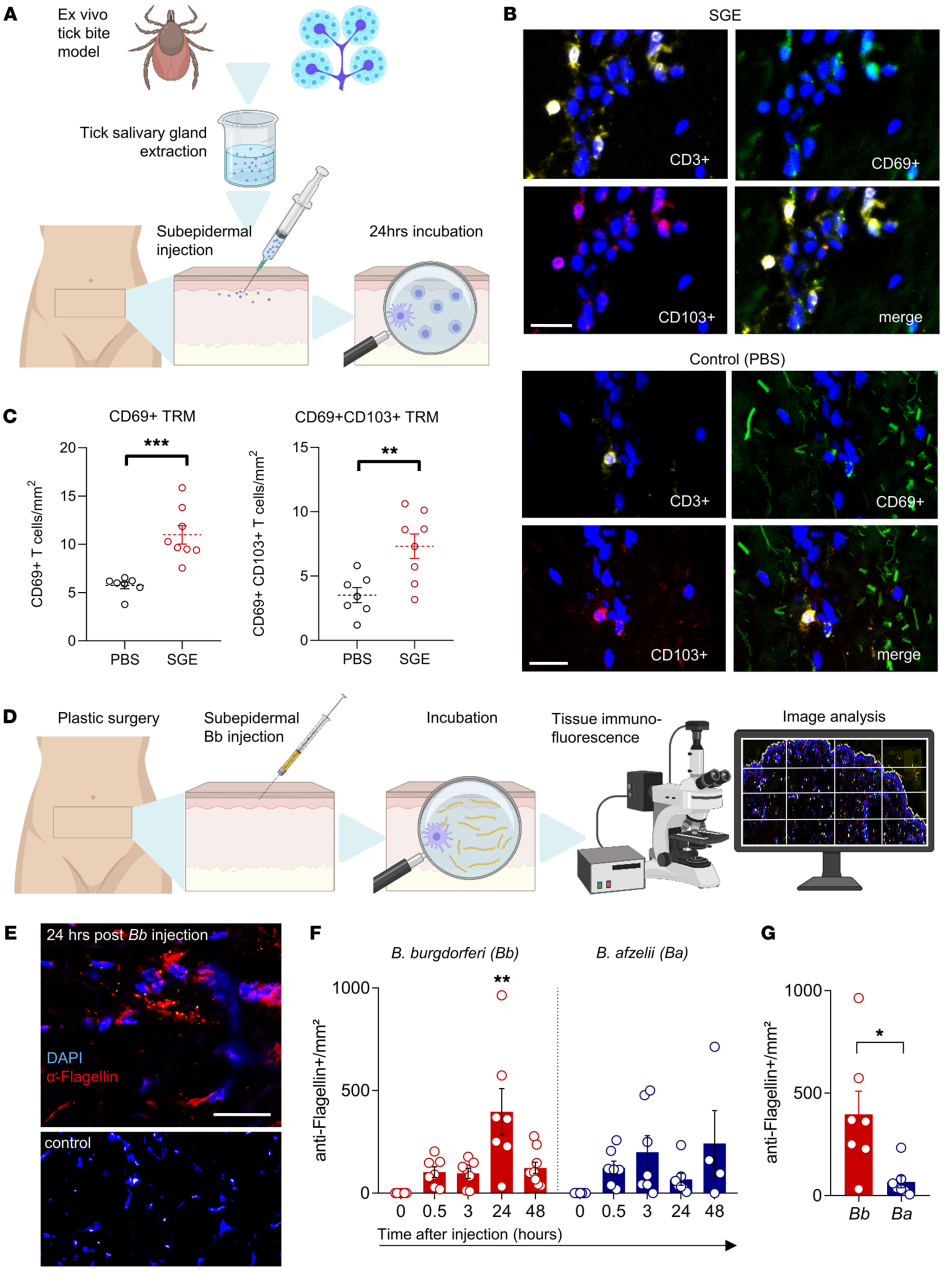
Early steps of tick-borne pathogen transmission are mimicked in an ex vivo human skin tick bite model. (**A**) Illustration of the ex vivo TB model in human skin. (**B**) Representative images of CD3-, CD69-, and CD103-immunolabeled and DAPI-counterstained skin injected with either SGE or PBS control. Scale bar: 20 μm. (**C**) CD69^+^ and CD69^+^CD103^+^ tissue-resident T cells per mm^2^ as determined by immunolabeling in skin injected with SGE (*n =* 8) or PBS (*n =* 7). (**D**) Illustration of the ex vivo *Bb* (*Borrelia* *burgdorferi* B31 strain) infection model. (**E**) Representative image of *Bbsl*-specific anti-flagellin and DAPI counterstaining in skin 24 hours after injection of *Bb* or culture media control. Scale bar: 50 μm. (**F**) Number of *Bbsl-*specific flagellin^+^ spirochetes per mm^2^ in skin cryosections before and after injection of *Bb* (*n =* 7) spirochetes or *Borrelia* *afzelii*, PKO strain (*Ba*, *n =* 7). Number of injected spirochetes: 1 × 10^5^/sample. Data shown as mean spirochete number/mm² at 0, 0.5, 3, 24, and 48 hours after injection. (**G**) Infection load (spirochetes per mm^2^) after *Bb* versus *Ba* injection. In **C**, **F**, and **G**, data are presented as mean ± SEM. Each dot represents the mean of 2 technical replicates. Statistical analysis was performed by unpaired Student’s *t* test (**C** and **G**) or 1-way ANOVA (**F**). *P < 0.05; **P < 0.01; ***P < 0.001.

Thus far, we found that tick feeding on human skin elicits several profound local and even systemic immunological changes, which may be important in transmission of tick-borne pathogens. However, models for tick feeding in human skin are urgently needed to investigate initial events at the host-pathogen interface and potential interventions. We therefore developed an experimental model for tick feeding on human skin (Figure 5A). The composition of tick saliva was analyzed extensively in many in vitro studies using tick saliva or salivary gland extract (SGE) (9–12). Tick saliva molecules mediating immunosuppression include the anticomplement molecules Salp20 and Isac (33, 34) that have been determined as basic proteins with a molecular weight of 30 to 36 kDa (35). We obtained SGE from *I*. *ricinus* ticks by using a technique similar to that described by Kim et al. (11). Subsequently, we injected the harvested SGE subepidermally into large full-thickness abdominal skin explants obtained from skin reduction surgeries. The skin samples were incubated in sterile media for 24 hours. We observed an increase in total T cell numbers after SGE injection (Supplemental Figure 5B), which corresponded to our results of biopsies from TB-affected individuals. Importantly, we also found significantly higher numbers of TRM-marker-expressing epidermal T cells in skin after SGE injection, arguing for rapid local transdifferentiation of nonresident T cells to TRMs by upregulation of residency factors (Figure 5, B and C, and Supplemental Figure 5C). During incubation, T cells displayed emigration rates into skin supernatants comparable to T cells of samples injected with PBS (Supplemental Figure 5D). In addition, the numbers of epidermal but not dermal LCs were reduced after injection of SGE (Supplemental Figure 5E). Owing to the comparable results obtained by analyzing natural TB sections in comparison to the subepidermal injection of tick SGE in explanted skin, we conclude that our method is a suitable model to study the human/tick host–vector interface ex vivo.

### Early steps of tick-borne pathogen transmission are mimicked in an ex vivo human skin infection model.

We adapted our ex vivo model to study the impact of *Borrelia* infection on cutaneous leukocytes (Figure 5D). As pathogens, we used the most common *Bbsl* strains, namely the *Bbss* strain B31 (*Bb*), originally isolated from *I*. *scapularis,* formerly *I*. *dammini*, USA (36), and *B*. *afzelii* strain PKO (*Ba*), originally isolated from human skin in Germany (37). We detected spirochetes by *Bbsl*-specific flagellar antigen immunofluorescent staining in all ex vivo–infected samples and noticed an increase in total numbers of detected spirochetes, arguing for local bacterial proliferation (Figure 5, E and F). In dose-response studies we injected increasing amounts of spirochetes, with larger numbers of injected *Bb* spirochetes resulting in increased bacterial skin load (Supplemental Figure 5F) and increased neutrophilic infiltrate (Supplemental Figure 5G).

Notably, injection of 1 × 10^5^ spirochetes and incubation for 24 hours resulted in the highest *Bb* spirochete numbers, which corresponds to the growth speed described in the literature (38). Interestingly, we observed differential infection dynamics after injection with *Ba* (Figure 5, F and G), the strain most prevalent in skin infection in central Europe (39, 40).

### Strain-specific differences are found in immune response to Borrelia infection.

**Figure 6:**
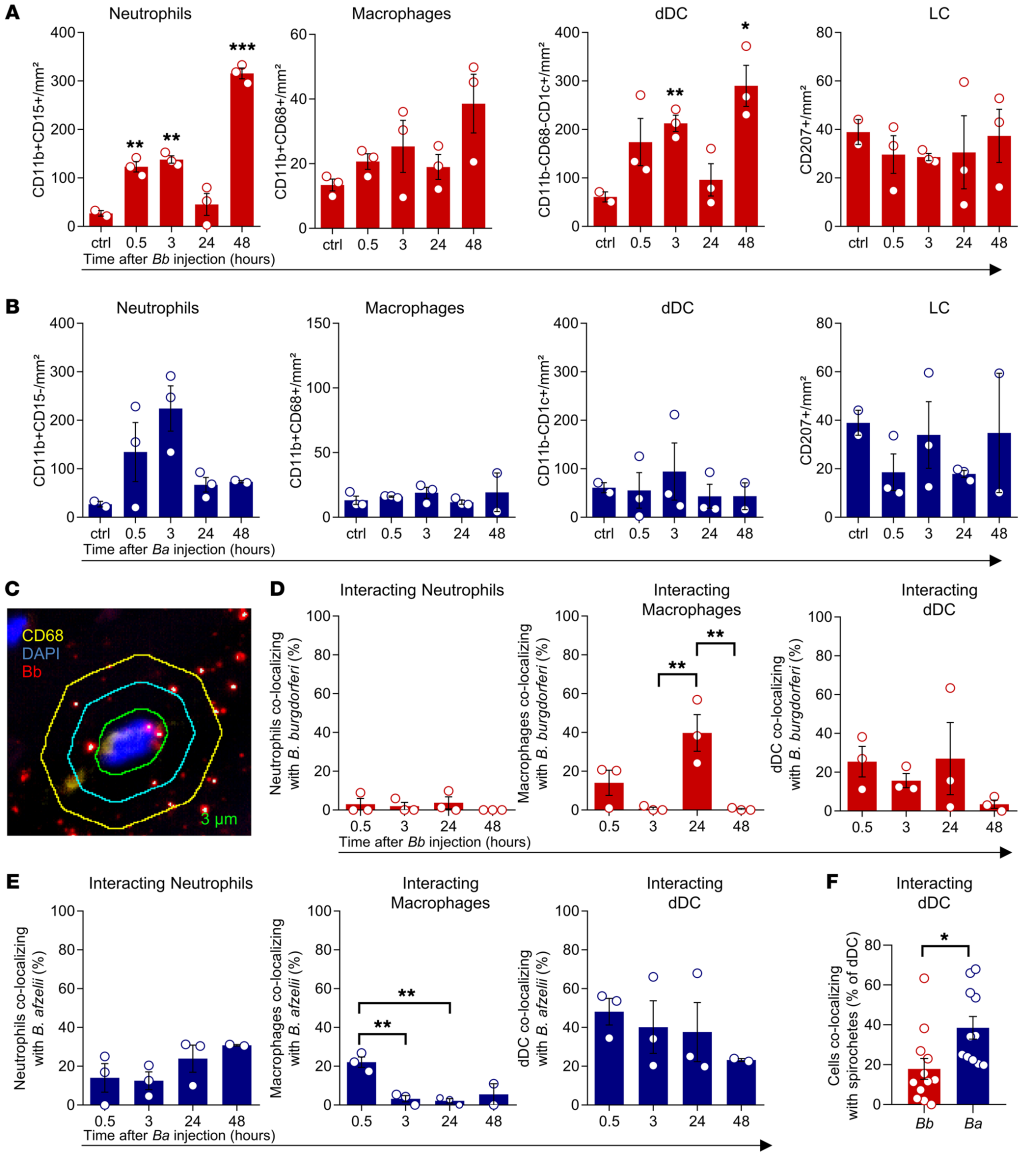
Early *Bb* model infection is accompanied by a strain-dependent influx of neutrophils and dDCs. (**A** and **B**) Percentage of neutrophils (CD11b^+^CD15^+^, *n =* 3), macrophages (CD15^–^CD11b^+^CD68^+^), dDCs (CD15^–^CD11b^–^CD11c^+^), and LCs (CD207^+^) in explanted abdominal skin after injection of *Bbsl* culture media (ctrl, *n =* 2) or *Bbsl* culture media containing *Bb* (**A**, *n =* 3) or *Ba* spirochetes (**B**, *n =* 3). Data shown as mean cell number/mm² 0.5, 3, 24, and 48 hours after injection and ctrl. Each dot represents the mean of 2 technical replicates. (**C**) Graphical representation of software-based analysis of cell-cell contact after immunostaining. Spirochetes (red/yellow dots) were analyzed for presence within green ring within 3 μm, indicating direct cell contact. (**D** and **E**) Percentages of neutrophils, macrophages, and dDCs colocalizing with spirochetes in skin explants injected with *Bb* (**D**, *n =* 3) or *Ba* (**E**, *n =* 3). Each dot represents the mean of 2 technical replicates. (**F**) Percentage of dDCs colocalizing with spirochetes after injection of *Bb* versus *Ba*. In **A**–**F**, data are presented as mean ± SEM. Statistical analysis was performed by 1-way ANOVA (**A**–**E**) or unpaired Student’s *t* test (**F**). *P < 0.05; **P < 0.01; ***P < 0.001.

Inferring that the presented human skin model provides the opportunity to study early events of the immune defense against *Bb* infection, we analyzed the immediate leukocyte response upon pathogen injection. As early as 30 minutes after injection, we observed increased numbers of neutrophils in samples injected with *Bb* (Figure 6A) and higher numbers of dDCs 3 hours after injection. No significant changes were detected in LC and macrophage numbers after *Bb* injection, and the changes occurring after *Ba* injection did not reach the level of significance (Figure 6B). To trace the initial steps of host-pathogen interaction, we next developed an image-based readout for cell-spirochete colocalization. Using custom-designed software, we calculated the distance between fluorescence-labeled spirochetes and surface-antibody-labeled immune cells using a ring mask identifier (Figure 6C). Relevant colocalization of *Bbsl* and immune cells that may allow interaction or represent pathogen uptake was defined as a 3 μm radius around the center of the cell nucleus. Independent of the *Bbsl* strain, macrophages and dDCs were often in close contact with spirochetes in the dermis at all time points studied, and likely constitute the primary cell subsets taking up pathogens and/or antigens (Figure 6, D and E). Surprisingly, we detected distinct strain-specific features of cell-pathogen colocalization; while in *Ba* infection most macrophage-spirochete colocalization events occurred early after injection (0.5 hours; Figure 6E), colocalization of *Bb* spirochetes and macrophages significantly increased after 24 hours (Figure 6D), which corresponds to the spirochete load reported above (Figure 5C). Furthermore, dDCs were more prone to colocalize with *Ba* spirochetes compared with *Bb* (Figure 6F). Both observations may explain superior infection control upon injection with *Ba*.

### Bb infection is modulated by tick SGE.

**Figure 7:**
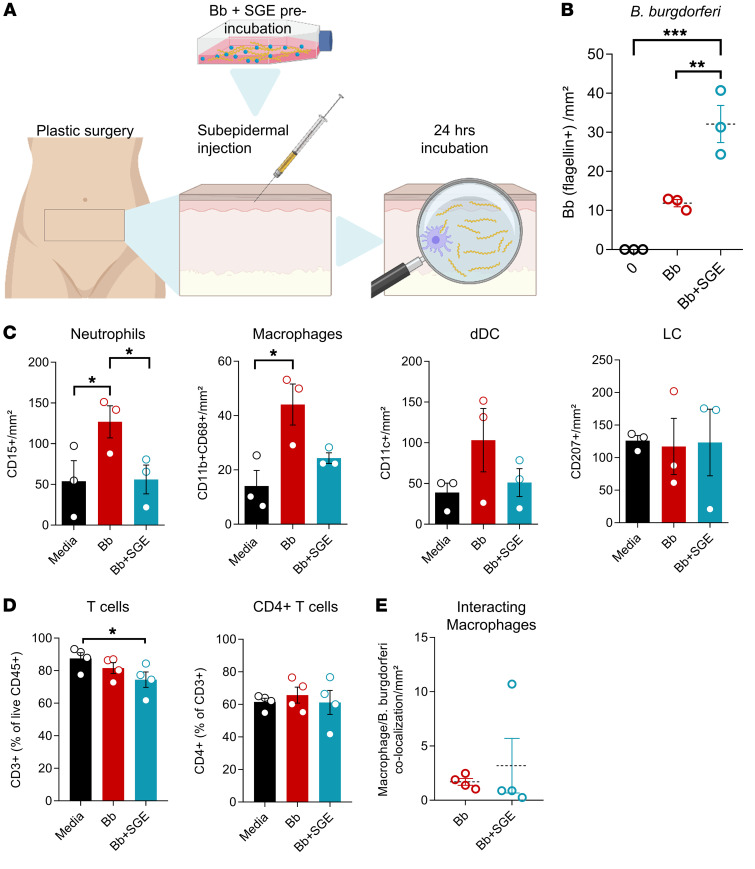
Preincubation of spirochetes with tick SGE dampens leukocyte response in the ex vivo skin model. (**A**) Illustration of the experimental model. (**B**) Number of spirochetes per mm^2^ in skin explants 24 hours after injection with media (0), *Bb*, or *Bb* preincubated with SGE (*Bb* + SGE). (**C** and **D**) Neutrophils, macrophages, dDCs, and LCs (**C**) and T cells and CD4^+^ T cells (**D**) per mm^2^ in skin explants injected with *Bb* (*n =* 3), *Bb* + SGE (*n =* 3), or cell culture media (*n =* 3) as determined by immunolabeling. (**E**) Percentage of macrophages colocalizing with spirochetes in skin explants 24 hours after injection with *Bb* or *Bb* + SGE. In **B**–**E**, data shown as individual data points, borders indicate mean, error bars indicate SEM. Statistical analysis was performed by 1-way ANOVA (**B**–**D**) or paired Student’s *t* test (**E**). *P < 0.05; **P < 0.01; ***P < 0.001.

Finally, we combined our 2 models to mimic incubation of *Bb* with tick salivary proteins in ticks prior to tick feeding (Figure 7A). For this purpose, we preincubated *Bb* with SGE for 15 minutes. When we injected the sample, we observed higher numbers of *Bb* in the dermis after preincubation (Figure 7B), indicating a pathogen-permissive microenvironment triggered by SGE. The addition of SGE caused a reduction in neutrophil and macrophage infiltration compared with *Bb* injection alone (Figure 7C). T cell numbers were reduced upon SGE and *Bb* introduction compared with mock media but not to *Bb* injection alone (Figure 7D). Furthermore, addition of SGE did not impair *Bb*-macrophage interactions (Figure 7E).

Overall, the presented results show an immunosuppressive effect of tick saliva components in the presence of *Bb* on neutrophils, lymphocytes, and macrophages, which are important cell types for the initiation of the immune response against infection.

## Discussion

Knowledge of complex immunomodulatory networks upon TB is pivotal for the successful application of so-called “tick vaccines,” which have recently been emerging as a breakthrough in tick-borne disease prophylaxis. An mRNA-based vaccine that encodes for 19 *I*. *scapularis* salivary proteins (19ISPs) was successful in impairing tick feeding and *B*. *burgdorferi* pathogen transmission in guinea pigs (41). Basophil clusters have been associated with this acquired resistance to tick feeding in mice and guinea pigs, which results in reduced size and numbers of engorged ticks upon reinfestation (42). Additionally, TRMs and mast cells are required for development of antitick immunity and CD4^+^ TRMs were increased after initial tick infestation in murine skin (43). We did not detect altered numbers of circulating or skin-bound basophils and mast cells in our cohort and patients reported no clinical indication of resistance to tick feeding. However, we did observe an increase in skin-resident memory T cells both in healthy individuals after tick feeding and in our ex vivo TB model, arguing for a similar TRM recruitment or transition in human skin of non–tick-resistant individuals.

In samples affected by TB, we detected increased cutaneous B cell frequencies. However, unlike in *Borrelia* infection, plasma cell levels remained unchanged after TB. This finding points to the role of B cells in immunosurveillance after skin barrier disruption and may indicate lack of Th cell signals for transdifferentiation to plasma cells after the bite of uninfected ticks. In line with this, simultaneously with a lymphocytic infiltrate consisting of CD8^+^ T cells, γδ T cells, TRMs, and B cells, we noticed significant impairment of T cell and ILC functions after TB. IL-17–producing ILC3s were diminished in skin and peripheral blood of TB-affected individuals. Interestingly, 19ISP mRNA vaccination induced IL-17 signaling pathways in guinea pigs (41), pointing toward the potential to reverse this tick-induced immunosuppression in the human host. In addition, we detected decreased percentages of IL-4–producing type 2 T cells and ILCs after TBs, an effect that may be mediated by iristatin contained in tick saliva, which has been found to inhibit IL-4 production in vitro (44). Saliva of the tick *Rhipicephalus microplus* suppressed Th1 cytokine production from peripheral blood mononuclear cells via the PD-1/PD-L1 pathway after feeding on cattle (45). We observed a reduction in IFN-γ production and type 1 responses in peripheral blood T cells and blood-derived and cutaneous ILCs, with a simultaneous decrease in circulating T cells and increase in skin TRMs. Therefore, a similar mechanism of T cell exhaustion might underlie the observed immunosuppression in human skin after *I*. *ricinus* feeding. Overall, systemic changes detectable in peripheral blood were limited, arguing for an effect primarily at the local TB site.

DCs were decreased in TB lesional skin and we were able to recapitulate the loss of epidermal LCs in an ex vivo TB model using *I*. *ricinus* SGE. The cause for reduced LC and dDC numbers remains to be investigated. Besides cell death, one potential explanation may be emigration to lymph nodes for antigen presentation, which could contribute to a tolerogenic environment after TB. In mice, it was shown that LC presence in regional lymph nodes skews the Th2 response after experimental tick infestation and LC-deficient mice displayed increased ability to develop Th1 responses upon *B*. *burgdorferi* infection (46). Future efforts in vaccine design should therefore focus on promoting an immunogenic DC response to induce a robust effector T cell immunity (47).

In the presented borreliosis model, we detected distinct modes of interaction with DCs for *Bb* and *Ba*. Macrophages showed high interactivity with *Bb* spirochetes, whereas dDCs were found close to *Ba* spirochetes. As the latter is more prevalent in Austria, one possible explanation could be previous exposure of skin donors to *Ba*. Interestingly, differences in cell-spirochete interactions correlated with species-specific infection cycles; spirochete load was significantly higher after infection with *Bb*, which is well known to cause more severe disease and a higher level of dissemination compared with *Ba* (48). Importantly, we were also able to mimic vector-bound *Bb* transmission by coincubation with tick SGE prior to injection in human skin. The additive effect of SGE resulted in decreased neutrophil, T cell, and macrophage numbers compared with infection without SGE, and increased spirochete load after incubation. However, this model is subject to limitations, as tick SGE may contain additional tick proteins not present in tick saliva, and salivary components are known to change over the course of feeding. Observed effects may thus be representative of one phase of the multivariable tick-feeding process.

The observed immunomodulation in human skin in response to TB and tick SGE likely influences not only spirochetal infection but also the course and likelihood of transmission of other pathogens. The here-described ex vivo human skin models offer a unique opportunity to study these initial steps of pathogen transmission at the vector-host interface. While we investigated the potential consequences for development of Lyme borreliosis, further studies are needed to address the immunomodulatory functions of tick saliva proteins in the context of other tick-borne diseases, including, but not limited to, tick-borne encephalitis, babesiosis, and rickettsiosis.

## Methods

### Study design and patient sampling.

We sampled healthy, adult individuals with a recent TB in medical history (≤9 days) after fully informed written consent. After local anesthesia with lidocaine hydrochloride (1% solution with epinephrine), 4 mm punch biopsies of the site of TB and healthy-appearing skin of the opposite limb or body site and blood samples were taken. Positive *Bbsl* detection resulted in sample exclusion. Full-depth human skin and subcutaneous adipose tissue samples 10 × 10 cm for the ex vivo skin model were obtained from abdominoplastic reduction surgeries performed at the Department of Plastic and Reconstructive Surgery, Medical University of Vienna.

### Sample processing and flow cytometry.

Isolated skin after enzymatic tissue digestion and peripheral blood mononuclear cells after Ficoll density gradient processing (Ficoll-Paque, VWR) were stained in single-cell suspensions by surface and intracellular markers, as previously described (49). Antibodies are listed in Supplemental Table 1. Single-cell suspensions were analyzed using FACSDiva software on a FACSAria III cell sorter (BD Biosciences) and FACS data were analyzed by FlowJo software (BD Biosciences).

### Cytokine secretion assays.

Cytokine release assays were performed upon stimulation with Cell Stimulation Cocktail (BioLegend) containing phorbol 12-myristate 13-acetate (PMA), ionomycin, and brefeldin A for 4 hours at 37°C. Cells were labeled using surface antibodies followed by fixation, permeabilization, and intracellular cytokine staining using the Intracellular Cytokine Staining kit (BioLegend) according to the manufacturer’s protocol.

### Tissue immunofluorescence.

Multicolor immunofluorescence staining of skin cryosections was performed with directly and indirectly labeled monoclonal antibodies (Supplemental Table 1). In brief, after incubation with the primary antibodies overnight, appropriate secondary fluorescently labeled antibodies were applied for 30 minutes at room temperature, followed by counterstaining with 4′,6-diamidino-2-phenylindol (DAPI). Immunostaining was controlled with isotype-matched conjugate antibodies. Images were acquired using a Z1 Axio Observer microscope equipped with a LD Plan-Neofluar 20×/0.4 objective (Zeiss) and quantified using TissueFaxs/TissueQuest and StrataQuest image analysis software (TissueGnostics).

### Ex vivo skin wounding model.

To investigate changes induced by sterile skin barrier disruption, we obtained 15 × 15 cm abdominal skin explants from plastic surgery procedures of healthy female donors upon appropriate fully informed written consent. Immediately after explant, samples were immersed in RPMI-1640 media (Gibco), followed by the infliction of a sterile wound using a 27 gauge hypodermic needle. Tissue was incubated with the needle in loco at 37°C for 24 hours. After incubation, a 6 mm punch biopsy was extracted from the wound and an unaffected control site 10 cm away from the puncture. Skin digestion was performed using collagenase IV and the gentleMACS (Miltenyi Biotec) dissociation program for human skin. Subsequently, single-cell suspensions were analyzed using flow cytometry as described above.

### Tick salivary gland preparation and handling.

Tick SGEs were obtained from the Slovak Academy of Sciences (Bratislava, Slovakia), and the preparation method was adapted from the previously described techniques of Slovák et al. and Kim et al. (50, 51). In brief, adult *I*. *ricinus* ticks were collected in non–TBE endemic regions of Slovakia. Ticks were fed on rabbits for 5 days, after which the ticks were gently removed and salivary glands were dissected, pooled in ice-cold PBS (Gibco), and homogenized. SGEs were stored and shipped at –80°C before further use.

### Ex vivo infection model.

We injected the 2 *Bbsl* strains (*Bb* B31 and *Ba* PKO laboratory strains) with and without tick SGE into full-thickness human skin explants. SGEs were injected at 20.6 μg/mL diluted in PBS supplemented with protease inhibitor cocktail (Merck). *Bb* spirochetes were from active B31 and PKO cultures in bacterial growth phase. Strains were cultured as previously described (52) at 34°C in a modified BSK II medium containing of gelatin solution and CMRL-1066 media (Sigma-Aldrich), supplemented with Neopeptone (Gibco), yeast extract, HEPES sodium salt (Sigma-Aldrich), glucose, sodium bicarbonate, sodium citrate dehydrate, sodium pyruvate, N-acetyl-D-glucosamine, 30% bovine serum albumin solution (Sigma-Aldrich), and heat-inactivated rabbit serum (R&D Systems) (52). Spirochetes were injected at a final concentration of 4 × 10^6^ bacteria/mL with or without preincubation for 15 minutes with SGE. Prior to injection, spirochetes were washed in 1× PBS and centrifuged at 8,000*g* for 20 minutes to remove culture media. Full-thickness skin flaps injected with *Bb*, *Ba*, *Bb* plus SGE, or media control were incubated in antibiotic-free sterile RPMI-1640 medium (Gibco) supplemented with 2% fetal bovine serum (Gibco) for 30 minutes, 3 hours, 24 hours, or 48 hours at 37°C. Biopsies from the injection site were taken, cryosectioned, and subjected to tissue immunofluorescence as described above. Image analysis was performed using custom-designed StrataQuest software (TissueGnostics) for the software-based measurement of (a) total cell number of immune cell types, (b) total number of *Bbsl* spirochetes, and (c) the distance of immune cells to *Bbsl* spirochetes in skin sections.

### Borrelia culture and PCR from skin samples.

To detect *Borrelia* in skin samples after TB, culture of *Bbsl* was attempted by inserting the allocated biopsy samples into 6 mL of modified BSK II medium as described above. The cultures were checked once a week by darkfield microscopy for spirochetal growth. Moreover, after 2 weeks a subculture was set up. After 8 weeks, DNA was extracted from 1.5 mL of culture to check for borrelial DNA.

The DNA from the biopsy samples and cultures were extracted using the Qiagen DNeasy Blood & Tissue Kit according to the manufacturer’s instructions. Screening for *Bbsl* was achieved by 2 real-time PCRs: one targeting the *16S* rDNA gene (53) and the other one the flagellin gene (54).

### Data and materials availability.

All data are available in the main text or the supplemental materials. Further information and requests for resources and reagents can be directed to the corresponding author.

### Statistics.

Statistical analyses were performed using Prism 8 (GraphPad Software). Statistical significance was determined by 2-tailed Student’s *t* test when comparing 2 groups and 2-way analysis of variance (ANOVA) when comparing 3 or more groups. Tukey’s multiple comparison post hoc test was used for multiple testing correction. Significance was set at a *P* value of less than 0.05.

### Study approval.

The study was approved by the Ethics Committee of the Medical University of Vienna (ECS 1281/2018) and was performed in accordance with the guidelines of the Declaration of Helsinki.

## Author contributions

JS conceptualized the study, developed methodology, and contributed to the investigation, data analysis, and writing of the original draft of the manuscript. VM, AMS, SM, and AG developed methodology and contributed to the investigation and data analysis. SB, LK, AEAG, SW, and DA contributed to the investigation and data acquisition. CS, AR, LU, RK, G Stary, and MM contributed to patient recruitment, clinical assessment, and sampling. G Stanek, MM, HS, and G Stary conceptualized and supervised the study and acquired funding. All authors revised the original draft of the manuscript.

## Supplementary Material

Supplemental data

## Figures and Tables

**Table 1:**
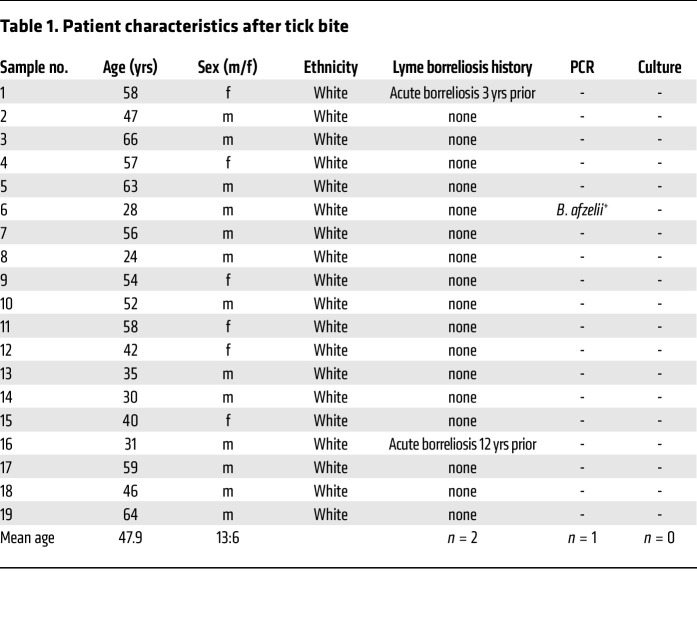
Patient characteristics after tick bite

## Version 1 09/27/2022

In-Press Preview

## Version 2 11/01/2022

Electronic publication
