# Supplementary material for: Tick feeding modulates the human skin immune landscape to facilitate tick-borne pathogen transmission
Source: J Clin Invest. 2022 Nov 1;132(21):e161188. doi: 10.1172/JCI161188 (PMC9621130; doi:10.1172/JCI161188)
Supplement: Supplemental data [file jci-132-161188-s053.pdf]

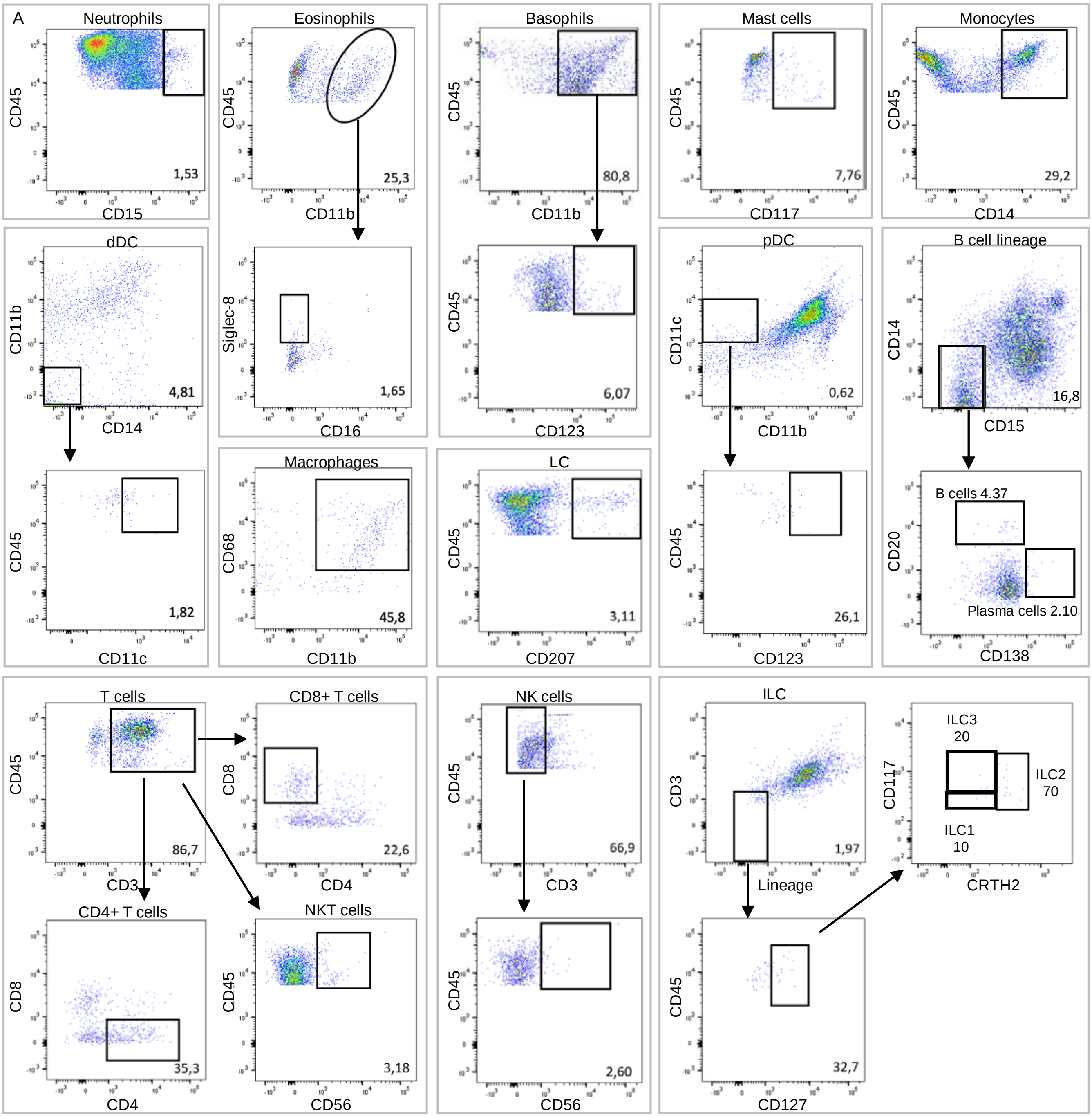

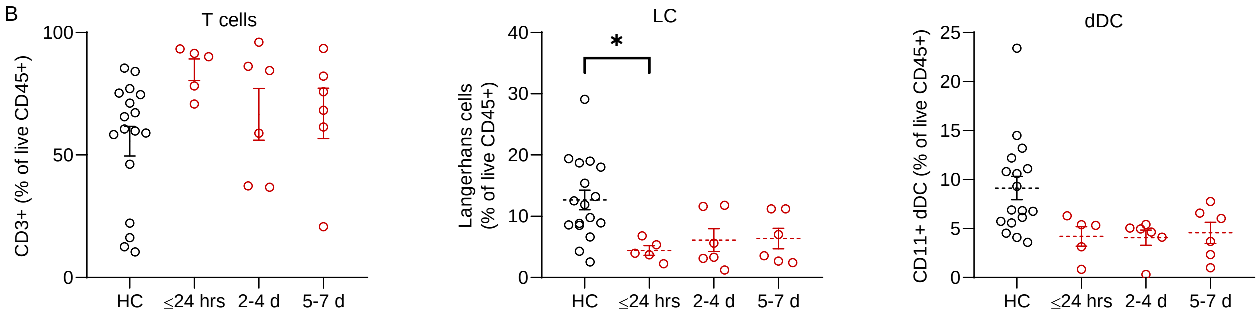

Supplementary Figure 1. Flow cytometric analysis of the cutaneous immune network upon tick bite (A) Representative gating strategies for neutrophils, eosinophils, basophils, mast cells, monocytes, macrophages, dDC, pDC, LC, B cells, plasma cells, T cells, CD4+ T cells, CD8+ T cells, NK cells, NKT cells, ILC and ILC subtypes from skin. **(B)** Percentage of T cells, LC and dDC among live cells in skin from HC site or TB site ≤24 hrs, 2-4 days or 5-7 days after TB. Error bars indicate SEM. Statistical analysis was performed by One-way ANOVA, * $p<0.05$.

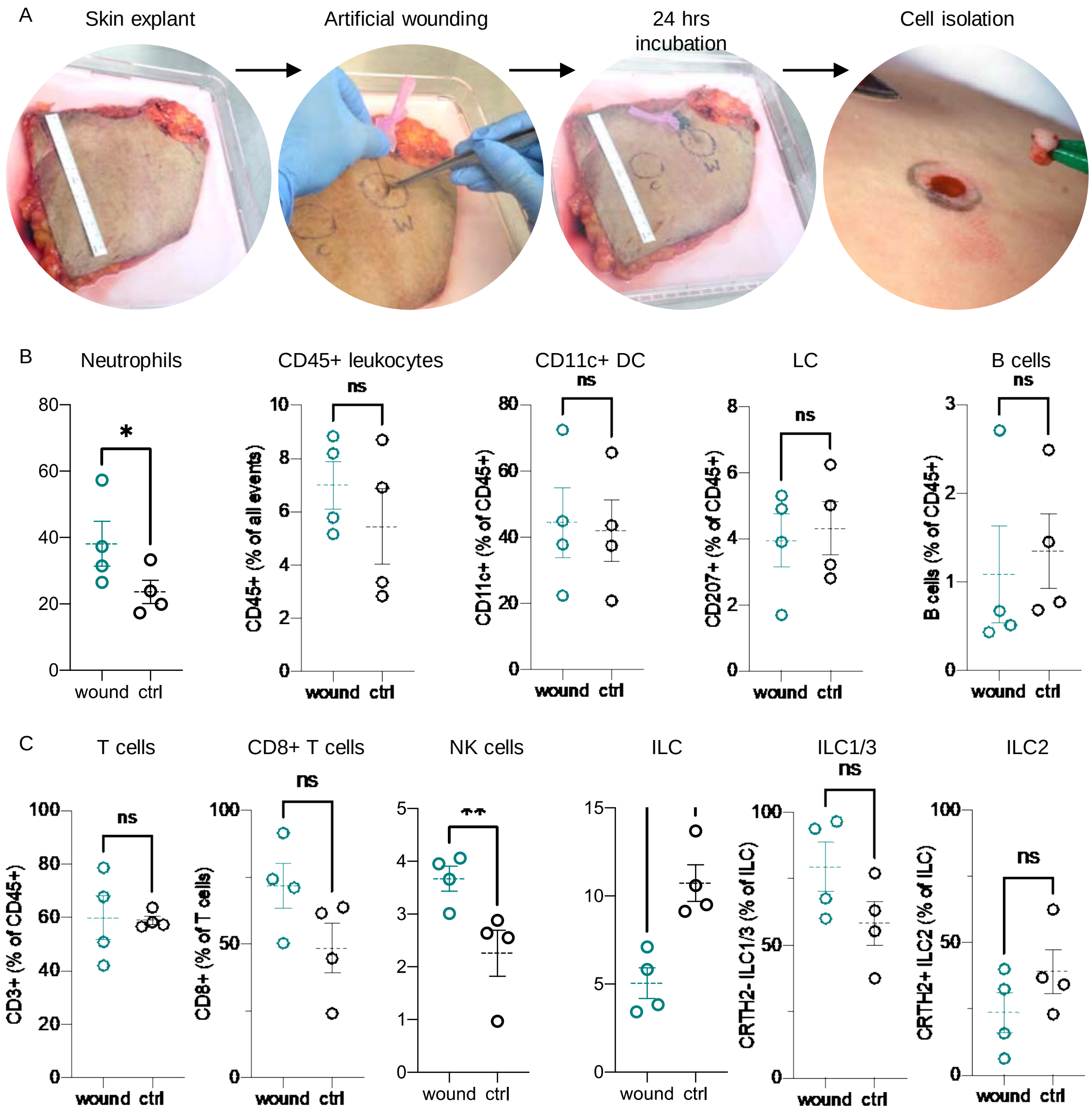

Supplementary Figure 2. Analysis of skin puncture sites. (A) Representative images of methodology for artificial wounding experiments using human skin explants. (B) Quantification of CD15+CD11b+ neutrophils, CD45+ leukocytes, CD11+ DC, CD207+ LC and B cells in artificially punctured skin ("wound") and matched control (non-wounded skin explant, "ctrl"). (C) Quantification of lymphocytes and lymphocyte subsets in samples from (A-B). Data shown as percentage of cells determined by flow cytometry. One dot represents one skin explant donor (mean of two technical replicates), n=4. Statistical analyses were performed using Student's t-test and correction for multiple testing, * $p<0.05$, ** $p<0.01$, ^{ns}, not significant.

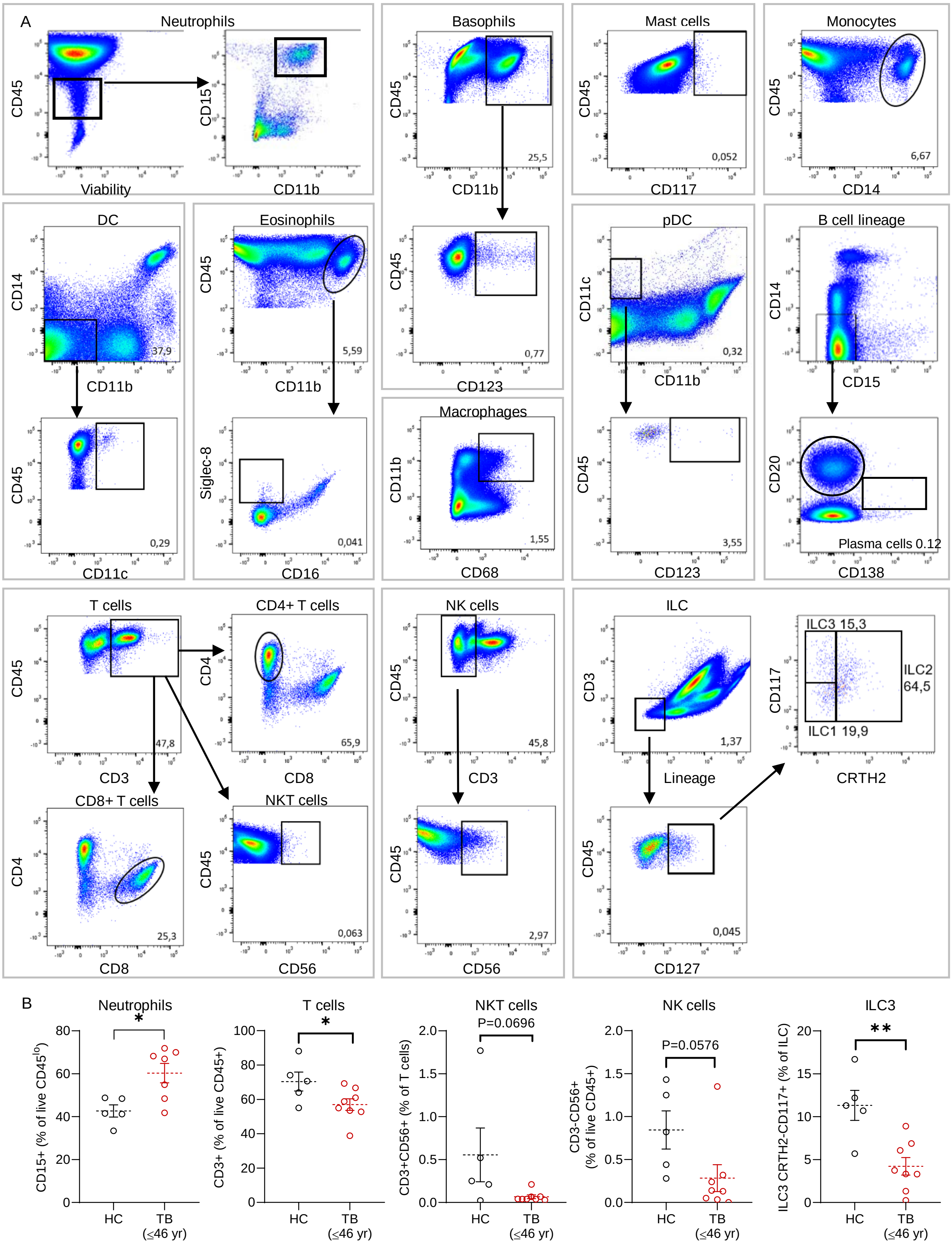

Supplementary Figure 3. Flow cytometry analyses of blood immune cells after cutaneous tick feeding. (A) Representative gating strategies for neutrophils, eosinophils, basophils, mast cells, monocytes, macrophages, DC, pDC, B cells, plasma cells, T cells, CD4+ T cells, CD8+ T cells, NK cells, NKT cells, ILC and ILC subtypes isolated from peripheral blood. **(B)** Analysis of age-matched HC and TB donors presented in Figure 2. Data shown as percentages of neutrophils, T cells, NKT cells, NK cells among live CD45+ cells and CRTH2-CD117+ (ILC3) among ILC in blood of HC (n=5) and young individuals affected by TB (age ≤46 years) (n=8). One dot represents one patient. Error bars indicate SEM. Statistical analysis was performed by unpaired Student's T-test, * $p<0.05$, ** $p<0.01$.

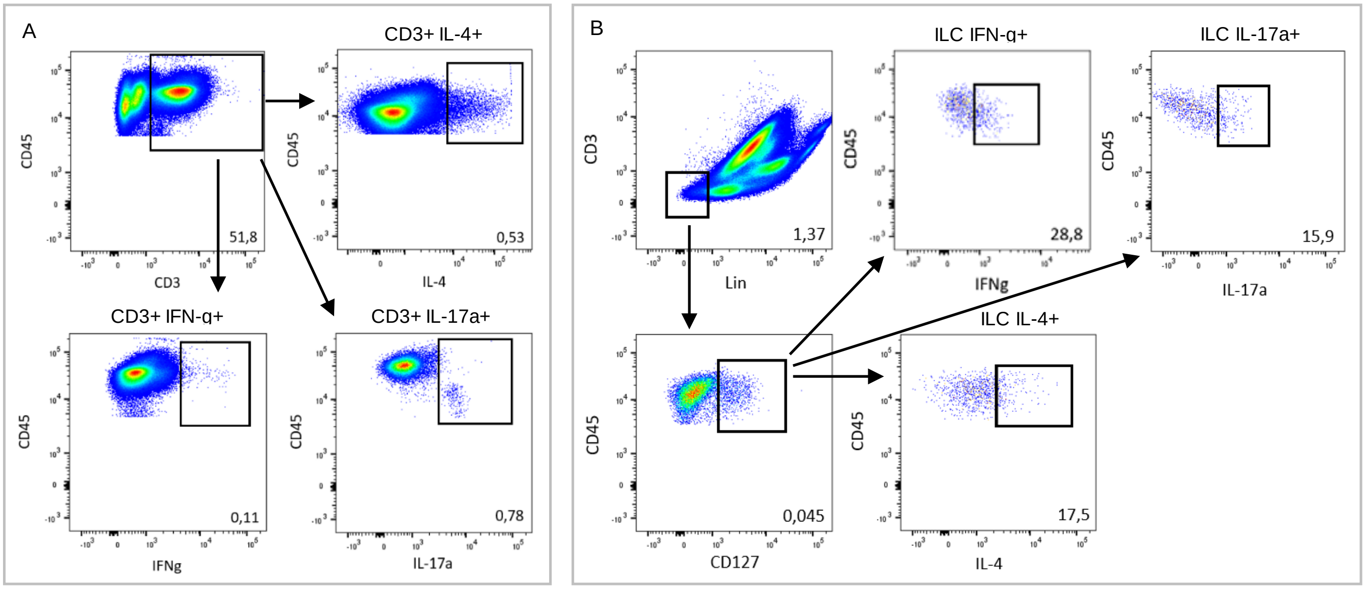

Supplementary Figure 4. Flow cytometry analyses of cytokine expression upon TB. Representative gating strategies for IFN-γ, IL-4 and IL-17a expressing T cells **(A)** and ILC **(B)** in blood and skin.

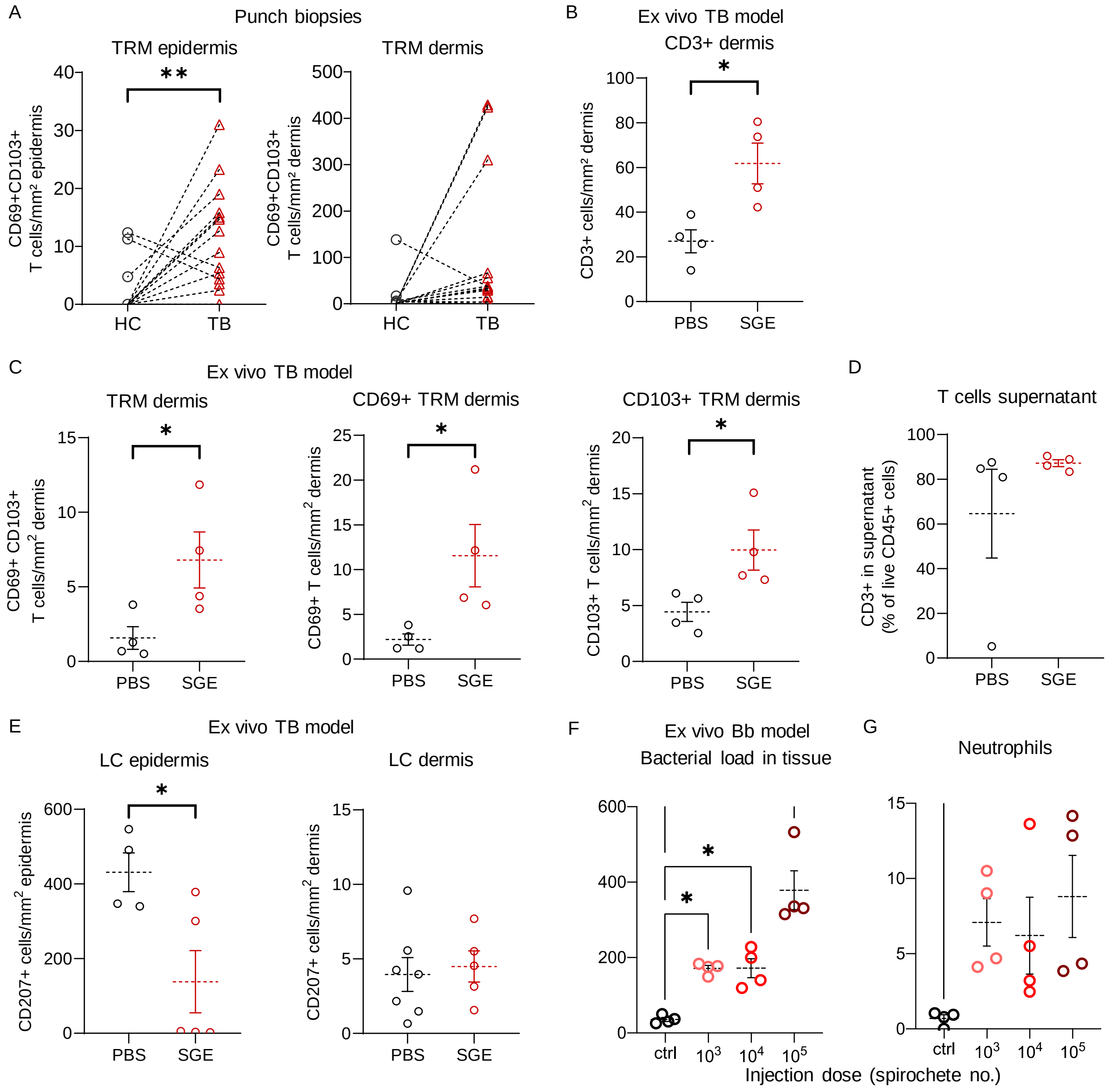

Supplementary Figure 5. Analysis of cellular responses to tick bite, SGE and Bb injection. (A) CD103+ and CD69+ double positive T cells per mm^2 as determined by immunolabeling in TB (n=11) and HC (n=11) punch biopsies. **(B-C)** CD3+ T cells **(B)** and CD69+/CD103+ TRM **(C)** per mm² dermis as determined by immunolabeling after injection of tick SGE (n=4) compared to PBS control (n=4). **(D)** Percentages of T cells in tissue supernatant after injection of SGE (n=4) or PBS control (n=4) determined by flow cytometry. **(E)** LC/mm² epidermis and dermis as determined by immunolabeling after injection of tick SGE (n=4) compared to PBS control (n=4). Data shown as mean cell numbers per mm^2 skin +/-SEM (A, B, C, E) and mean percentage of live CD45+ cells in supernatant (D). **(F-G)** Dose-response study of the ex vivo Bb model. Data shown as Bb-specific flagellar AG-positive structures/mm² (F) and CD15+CD11b+DAPI+ cells/mm² skin 24 hrs after subepidermal injection of 1x10^3, 1x10^4 or 1x10^5 Bb spirochetes in human skin explants and incubation at 37°C (n=4, mean of two technical replicates +/- SEM). Statistical analysis was performed by paired (A) or unpaired (B-F) Student's T test and one-way ANOVA with Turkey's post-test (F,G), * $p<0.05$, ** $p<0.01$, *** $p<0.001$.
